# Cholera in Chicago

**Published:** 1850-10

**Authors:** 


*Cholera in Chicago.*—We clip from the *Daily Journal* the following statement of the total mortality, presuming that it is as nearly as possible correct:—

"The total number of deaths in July, 240; total in August, 466.

Deaths from Cholera from the 23d of June, the time when the epidemic made its appearance, to the 1st of August, 158; from the 1st of August, to 1st of September, 283, making a total of deaths by Cholera during the season, of 441.

Thus the whole mortality of the city, from the 23d of June to the 1st of September, a period of 69 days, is not far from 624, making an average of about nine per day, which, taking the population at 27,000, gives one death daily to every 3,000."

